# Intimate partner violence, depression, hazardous alcohol use, and social support among people with HIV initiating HIV care in Cameroon

**DOI:** 10.1371/journal.pone.0304114

**Published:** 2024-05-21

**Authors:** Kathryn E. L. Grimes, Peter Vanes Ebasone, Anastase Dzudie, Denis Nash, Brian W. Pence, Milton Wainberg, Marcel Yotebieng, Rogers Ajeh, Angela M. Parcesepe

**Affiliations:** 1 Department of Health Policy and Management, Gillings School of Global Public Health, University of North Carolina at Chapel Hill, Chapel Hill, NC, United States of America; 2 Clinical Research Education Networking and Consultancy, Yaoundé, Cameroon; 3 Institute for Implementation Science in Population Health, City University of New York, New York, NY, United States of America; 4 Department of Epidemiology, Gillings School of Global Public Health, University of North Carolina at Chapel Hill, Chapel Hill, NC, United States of America; 5 Department of Psychiatry, Columbia University, New York, NY, United States of America; 6 New York State Psychiatric Institute, New York, NY, United States of America; 7 Department of Medicine, Albert Einstein College of Medicine, Bronx, NY, United States of America; 8 Department of Maternal and Child Health, Gillings School of Global Public Health, University of North Carolina at Chapel Hill, Chapel Hill, NC, United States of America; 9 Carolina Population Center, University of North Carolina at Chapel Hill, Chapel Hill, NC, United States of America; South African Medical Research Council (SAMRC) / Stellenbosch University (SU), SOUTH AFRICA

## Abstract

Intimate partner violence (IPV) has been associated with poor mental health among people with HIV (PWH) globally. Social support may be a strategy to foster mental health among PWH. Little is known about whether the relationship between IPV and mental health differs by IPV type or level of social support. Interviews were conducted with 426 PWH initiating HIV care in Cameroon. Log binomial regression analyses were used to estimate the association between four types of IPV (controlling behavior and emotional, physical, and sexual IPV) and symptoms of depression or hazardous alcohol use, separately by IPV type and level of social support. Over half (54.8%) of respondents experienced moderate/high levels of controlling behavior, 42.0% experienced emotional IPV, 28.2% experienced physical IPV and 23.7% experienced sexual IPV. Controlling behavior was associated with greater prevalence of depressive symptoms. This relationship did not vary meaningfully by level of social support (low: aPR 2.4 [95% CI 1.2, 4.9]; high: 1.7 [95% CI 1.0, 2.7]). Emotional and physical IPV were associated with greater prevalence of depressive symptoms among those with low social support (emotional IPV: aPR 1.9 [95% CI 1.0, 3.4]; physical IPV: aPR 1.8 [95% CI 1.2, 2.8]), but not among those with high social support (emotional IPV: aPR 1.0 [95% CI 0.7, 1.6]; physical IPV: aPR 1.0 [95% CI 0.6, 1.6]). Controlling behavior, emotional IPV, and physical IPV were associated with a greater prevalence of hazardous alcohol use, with moderately larger effect estimates among those with high compared to low social support. Sexual IPV was not associated with depressive symptoms or hazardous alcohol use. Services to screen and care for people experiencing IPV are urgently needed among PWH in Cameroon. Future research to identify barriers, feasibility, acceptability, and organizational readiness to integrate IPV and mental health services into HIV care settings is needed.

## Introduction

Intimate partner violence (IPV) is a global health issue affecting approximately one third of women worldwide [1]. The World Health Organization defines IPV as violence occurring within the context of a current or past intimate relationship, “including acts of physical aggression, sexual coercion, psychological abuse and controlling behaviors” [2]. Globally, an estimated 27% of ever-partnered women have experienced sexual or physical IPV in their lifetime and 13% have experienced such violence in the past year [3]. IPV has also been commonly reported among people with HIV (PWH) across global settings. A meta-analysis estimated that 40% of PWH globally have ever experienced some type of IPV (physical, sexual, emotional, or psychological IPV) and 20% have experienced IPV in the past year, with emotional IPV the most prevalent type of IPV reported [4]. A separate meta-analysis estimated that 33% of women with HIV in sub-Saharan Africa have experienced IPV [5].

IPV has been consistently associated with adverse physical outcomes including injury and death, among others. Among PWH, IPV has also been associated with poor HIV treatment outcomes throughout the HIV care continuum, including delayed ART initiation, suboptimal ART adherence, and unsuppressed HIV viral load [6–9]. IPV has been associated with poor mental health among both the general population and among PWH. Among the general population, IPV has been associated with increased prevalence of depression, post-traumatic stress disorder (PTSD), anxiety, and harmful alcohol use [1]. Among women with HIV, IPV has been associated with increased prevalence of depression, anxiety, and heavy drinking [10–12]. IPV intensity (i.e., severity, frequency) has been found to increase the likelihood of mental health comorbidities, such as co-occurring PTSD and depression [13].

Mental health disorders are more common among PWH compared to the general population [14]. It has been estimated that approximately half of PWH have a mental health disorder, with depression being the most prevalent [14]. A meta-analysis estimated that between 14%-32% of PWH on antiretroviral treatment (ART) in sub-Saharan Africa experienced depression [15]. Additional studies have estimated depression prevalence among adults on ART to be between 18–25% [16, 17]. Alcohol use frequently co-occurs with depression [18]. Alcohol is the most commonly used substance throughout sub-Saharan Africa and commonly used by PWH. A meta-analysis estimated the pooled prevalence of alcohol use disorder among PWH to be 30% [19], and a study among PWH initiating ART in Uganda and South Africa found 32% exhibited biomarker-measured unhealthy alcohol use [20]. Prior research suggests that relationships between IPV and both depression and alcohol use are bidirectional, with IPV associated with subsequent depression and alcohol use, and depression and alcohol use associated with subsequent IPV [21, 22], though further research is needed to better elucidate these relationships, especially among PWH.

Despite consistent associations between IPV and poor mental health, little is known about whether this relationship differs by type of IPV experienced. To date, most research on the relationship between mental health and IPV has focused on sexual and physical IPV. However, emotional and psychological violence are often the most prevalent types of IPV experienced among PWH [4, 5]. Further, among studies that have examined multiple types of IPV, emotional and psychological IPV have been found to have stronger associations with depression and substance use [23, 24] compared to physical or sexual IPV. Additionally, psychological abuse and stalking have been found to be independently associated with PTSD and depression, even after controlling for sexual and physical IPV [25].

Social support may be a strategy to foster mental health among PWH. Social support has been found to be associated with better overall health [26, 27], health-related quality of life [28], and mental health among PWH. Among PWH in Namibia and Ethiopia, social support has been found to be inversely associated with psychological distress and depressive symptoms [29, 30]. Findings on the relationship between social support and alcohol use remain equivocal. While some studies among PWH found no association between social support and alcohol use, other studies with PWH have found greater social support to be associated with greater unhealthy drinking [31–34]. Little is known about the extent to which social support modifies the relationship between IPV and mental health among PWH.

Greater understanding of the interrelationships among IPV, depression, hazardous alcohol use, and social support can inform the development and implementation of strategies to prevent and address IPV and improve the mental health and quality of life of PWH who have experienced IPV. This paper’s objectives are to (1) estimate the prevalence of four types of IPV (controlling behavior and emotional, physical, and sexual IPV) among PWH initiating HIV care in Cameroon; (2) assess the relationships among IPV, depression, and hazardous alcohol use; and (3) identify the extent to which the relationships among IPV, depression, and hazardous alcohol use vary by IPV type or level of social support.

## Methods

### Setting and study design

This observational, cross-sectional study was conducted in three urban HIV treatment facilities in Cameroon. These sites were selected because they participate in the International epidemiology Databases to Evaluate AIDS (IeDEA) Consortium [35]. Research assistants fluent in French and English conducted in-person structured interviews with participants between June 15, 2019 and March 13, 2020. The interview included questions on sociodemographics, mental health, alcohol use, IPV, and social support. The survey instrument was translated into French, back translated into English, and then pilot tested with key stakeholders prior to study implementation.

### Participants and recruitment

Individuals were eligible to participate if they were newly enrolling in HIV care at one of the three study facilities and were aged 21 or older. Participants under 21 years of age were excluded, as the Cameroonian government and the Cameroonian National Ethics Committee define 21 as the age of adulthood for individuals in Cameroon. Individuals were not eligible to participate if they were transferring HIV care from another facility. Participants were recruited by research staff at the study sites at the time of their HIV care appointment.

### Measures

#### Depressive symptoms

Depressive symptoms were assessed with the Patient Health Questionnaire-9 (PHQ-9) [36], which has been previously validated for use in sub-Saharan Africa among PWH [37–39]. This 9-item screener asks respondents about depressive symptoms in the previous 2 weeks. Scores of 10 or higher were categorized as moderate or severe depressive symptoms [36]. Cronbach’s alpha in this sample was 0.81.

#### Hazardous alcohol use

Alcohol use was assessed with the Alcohol Use Disorders Identification Test (AUDIT) [40]. This 10-item screener asks participants about alcohol use over the previous 12 months. AUDIT scores of 7 or greater for women and 8 or greater for men were categorized as hazardous alcohol use [41]. The AUDIT has been validated for use in sub-Saharan African populations [42, 43]. Cronbach’s alpha in this sample was 0.85.

#### IPV

The National Demographic and Health Survey (NDHS) [44] IPV module was used to assess experiences of IPV and included 4 IPV domains: controlling behavior, emotional IPV, physical IPV, or sexual IPV. Eighteen items asked participants how often their spouse or partner behaved in certain ways in the past year. Interviewers were trained to adapt questions as needed to ask in the past tense if the participant was thinking about a past relationship (e.g., “in the last year that you were together, how often…”). If the participant responded *yes* to at least one IPV item, they were categorized as having experienced IPV. To our knowledge, the NDHS IPV module has not been validated for use in Cameroon. However, a global measurement-invariance assessment found the physical IPV and controlling behavior items to be approximately invariant across 36 low- and middle-income countries, 22 of which were in sub-Saharan Africa [45].

#### Controlling behavior

Controlling behavior was assessed with five questions about whether the participant’s partner: *was jealous or angry if they talked to other men/women*, *frequently accused them of being unfaithful*, *did not permit them to meet friends*, *tried to limit their contact with family*, or *insisted on knowing where they were at all times*. Participants who responded *yes* to none or one of these questions were categorized as having experienced no or low levels of controlling behavior. Participants who responded *yes* to two or more questions were categorized as having experienced moderate or high levels of controlling behavior.

#### Emotional IPV

Emotional IPV was assessed with three items about whether the participant’s partner had: *done or said something to humiliate them*, *threatened them or someone close to them with harm*, or *insulted/belittled them*. Participants who responded *yes* to at least one of these questions were classified having experienced emotional IPV.

#### Physical IPV

Physical IPV was assessed with eight questions about whether the participant’s partner had: *pushed or thrown something at them*, *slapped them*, *twisted their arm or pulled their hair*, *punched them with a fist or something that could be harmful*, *kicked or dragged them*, *tried to strangle or burn them*, *threatened them with a knife*, *gun*, *or other weapon*, or *attacked them with a knife*, *gun*, *or other weapon*. Participants who responded *yes* to one or more of these items were classified as having experienced physical IPV.

#### Sexual IPV

Sexual IPV was assessed with two questions about whether the participant’s partner had: *physically forced them to have sexual intercourse* or *forced them to perform other sexual acts when they did not want to*. Participants who responded *yes* to either question were classified as having experienced sexual IPV.

#### Social support

Social Support was assessed with 4 survey items from the Multidimensional Scale of Perceived Social Support [46]: the participant *can count on friends when things go wrong*, *has friends with whom they can share joys and sorrows*, *gets emotional help and support needed from family*, or *can talk about problems with family*. Participants responded how much they agreed or disagreed with each statement on a 5-point Likert scale, with total possible scores ranging from 4 to 20. Scores were dichotomized at the midpoint, with participants categorized as having low (4–11) or high (12–20) social support.

#### Sociodemographic characteristics

Sociodemographic characteristics were self-reported and included gender, age, education, religion, relationship status, number of living children, employment status, time away from home, and household hunger. Household hunger was assessed using the Household Hunger Scale [47], which asks three questions about household hunger in the past four weeks (e.g., *In the past four weeks*, *was there ever no food to eat of any kind in your house because of lack of resources to get food*?). If participants responded affirmatively to any question they were asked a follow-up question about how frequently it occurred (response options were rarely [1–2 times], sometimes [3–10 times] or often [more than 10 times]). Responses indicating the event never occurred were coded as 0, rarely or sometimes occurred were coded as 1, and often occurred coded as 2, for an overall range of possible scores from 0–6. Scores of 2 or greater were classified as moderate or severe household hunger.

### Analysis

Univariate analyses were conducted to describe IPV prevalence. Bivariate analyses using Pearson chi-squared tests were used to assess the relationships among IPV and social support, depressive symptoms, and hazardous alcohol use. Model building was guided by directed acyclic graphs (DAGs) and informed by existing literature on potential confounders and mediators of the exposure-outcome relationships being assessed. Log-binomial regression analyses were used to assess the relationship between IPV and depression and hazardous alcohol use, separately, overall, and by level of social support (low vs. high). Adjusted models controlled for gender (male/female), relationship status (single/partnered), and clinic. Breusch-Pagan and Cook-Weisberg tests revealed heteroskedasticity issues; thus, regression models used robust standard errors. All statistical analyses were conducted on Stata version 17.0 [48].

### Ethical approvals

All participants provided written informed consent. Ethical approval for this study was obtained from the University of North Carolina’s Institutional Review Board and from the National Ethical Committee of Research for Human Health in Yaoundé, Cameroon.

## Results

A total of 426 participants completed interviews. Participants who reported having never been in an intimate relationship (*n* = 14) or had missing depression, alcohol use, or IPV data (*n* = 4) were removed from this analysis for an analytic sample of 405. Of the 405 individuals included in this analysis, a majority were women (57.8%) with a median age of 37 (interquartile range: 30–45) (Table 1). Most participants were currently in a relationship (59.5%) and working for pay (65.2%).

**Table 1:** Demographic characteristics and intimate partner violence among PWH initiating HIV care in Cameroon.

	Total (*N* = 405) *n*(col%)	Reported IPV Experience	
		No (*n* = 60) *n*(col%)	Yes (*n* = 345) *n*(col%)
Gender			
Male	171(42.2)	27(45.0)	144(41.7)
Female	234(57.8)	33(55.0)	201(58.3)
Age (median/IQR)	37 (30–45)	37 (31–46)	37 (30–45)
21–39	232(57.3)	32(53.3)	200(58.0)
40+	173(42.7)	28(46.7)	145(42.0)
Education			
None	29(7.2)	5(8.3)	24(7.0)
Primary	210(51.9)	28(46.7)	182(52.8)
≥Secondary	166(41.0)	27(45.0)	139(40.3)
Religion			
Catholic	148(36.5)	22(36.7)	126(36.5)
Protestant	135(33.3)	16(26.7)	119(34.5)
Born again	86(21.2)	19(31.7)	67(19.4)
Other	36(8.9)	3(5.0)	33(9.6)
Relationship status			
Single	164(40.5)	20(33.3)	144(41.7)
Partnered	241(59.5)	40(66.7)	201(58.3)
Number of living children^a^			
0	73(18.1)	13(21.7)	60(17.5)
≥1	330(81.9)	47(78.3)	283(82.5)
Employment status			
Not working for pay	141(34.8)	20(33.3)	121(35.1)
Working for pay	264(65.2)	40(66.7)	224(64.9)
Away from home >1 month in past year			
No	250(61.7)	39(65.0)	211(61.2)
Yes	155(38.3)	21(35.0)	134(38.5)
Household Hunger^a^			
No/low	293(72.7)	46(76.7)	247(72.0)
Moderate/Severe	110(27.3)	14(23.3)	96(28.0)

^a^Missing: Number of living children n = 2; Household hunger n = 2

IPV was commonly reported among participants. Most participants (85.2%) reported having experienced at least one form of IPV (84.2% of men and 85.9% of women). Over half (54.8%) reported having experienced moderate or high levels of controlling behavior, 42.0% experienced emotional IPV, 28.2% experienced physical IPV and 23.7% experienced sexual IPV (Table 2). More than half (53.8%) of study participants experienced emotional, physical, or sexual IPV. Overall, 19.5% reported moderate or severe depressive symptoms and 39.8% reported hazardous alcohol use (Table 2).

**Table 2:** IPV among PWH initiating care in Cameroon, by depressive symptoms, hazardous alcohol use, and social support.

	Total (N = 405) *n*(col%)	Depressive Symptoms			Hazardous Alcohol Use			Social Support*		
		No (*n* = 326)	Yes (*n* = 79)	p-value	No (*n* = 244)	Yes (*n* = 161)	p-value	Low social support (*n* = 98)	High social support (*n* = 305)	p-value
		**n (row%)**	**n (row%)**		**n (row%)**	**n (row%)**		**n (row%)**	**n (row%)**	
Controlling Behavior										
None/Low	183(45.2)	162(88.5)	21(11.5)	<0.001	119(65.0)	64(35.0)	0.074	43(23.6)	139(76.4)	0.769
Moderate/High	222(54.8)	164(73.9)	58(26.1)		125(56.3)	97(43.7)		55(24.9)	166(75.1)	
Emotional IPV										
No	235(58.0)	198(84.3)	37(15.7)	0.025	154(65.5)	81(34.5)	0.011	54(23.0)	181(77.0)	0.459
Yes	170(42.0)	128(75.3)	42(24.7)		90(52.9)	80(47.1)		44(26.2)	124(73.8)	
Physical IPV										
No	291(71.9)	239(82.1)	52(17.9)	0.184	177(60.8)	114(39.2)	0.704	67(23.2)	222(76.8)	0.398
Yes	114(28.2)	87(76.3)	27(23.7)		67(58.8)	47(41.2)		31(27.2)	83(72.8)	
Sexual IPV										
No	309(76.3)	252(81.6)	57(18.4)	0.334	189(61.2)	120(38.8)	0.498	68(22.1)	240(77.9)	0.059
Yes	96(23.7)	74(77.1)	22(22.9)		55(57.3)	41(42.7)		30(31.6)	65(68.4)	

*Missing: Social support (*n* = 2)

In bivariate analyses, having experienced moderate or high controlling behavior was significantly associated with greater prevalence of depressive symptoms, but not hazardous alcohol use (Table 2). Overall, 26.1% of those who experienced moderate or high controlling behavior reported moderate to severe depressive symptoms compared to 11.5% of those who experienced no or low levels of controlling behavior. In bivariate analyses, having experienced emotional IPV was associated with greater prevalence of depressive symptoms and hazardous alcohol use. Overall, 24.7% of those who experienced emotional IPV reported moderate to severe depressive symptoms compared to 15.7% of those who had not experienced emotional IPV. Similarly, 47.1% of those who experienced emotional IPV reported hazardous alcohol use compared to 34.5% of those who did not report experiencing emotional IPV. Having experienced sexual or physical IPV was not associated with depressive symptoms or hazardous alcohol use in bivariate analyses.

Among the entire sample, in multivariable analyses, compared to having experienced no or low levels of controlling behavior, having experienced moderate or high levels of controlling behavior was associated with greater prevalence of depressive symptoms (adjusted prevalence ratio [aPR] 1.9 [95% CI 1.2, 2.9]) and hazardous alcohol use (aPR 1.3 [95% CI 1.0, 1.7]) (Table 3). Among the entire sample, emotional IPV was associated with greater prevalence of hazardous alcohol use (aPR 1.5 [95% CI 1.2, 1.9]), but not depressive symptoms (aPR 1.2 [95% CI 0.8, 1.7]). Sexual and physical IPV were not associated with depressive symptoms or hazardous alcohol use in multivariable models in the entire sample.

**Table 3:** Bivariate and multivariable models of IPV, depressive symptoms, and hazardous alcohol use among PWH initiating care in Cameroon (*N* = 405).

	Depressive Symptoms				Hazardous Alcohol Use			
	PR (95% CI)	p-value	aPR (95% CI)*	p-value	PR (95% CI)	p-value	aPR (95% CI)*	p-value
Controlling Behavior	2.28 (1.44, 3.60)	<0.001	1.88 (1.23, 2.89)	0.004	1.25 (0.98, 1.60)	0.078	1.31 (1.04, 1.66)	0.023
Emotional IPV	1.57 (1.06, 2.33)	0.026	1.19 (0.83, 1.71)	0.338	1.37 (1.08, 1.73)	0.010	1.51 (1.21, 1.89)	<0.001
Physical IPV	1.33 (0.88, 2.00)	0.180	1.16 (0.80, 1.68)	0.442	1.05 (0.81, 1.37)	0.702	1.25 (0.97, 1.59)	0.079
Sexual IPV	1.24 (0.80, 1.92)	0.328	1.19 (0.81, 1.75)	0.383	1.1 (0.84, 1.44)	0.491	1.18 (0.91, 1.53)	0.220

PR: prevalence ratio; aPR: adjusted prevalence ratio

*Adjusted for gender, clinic, and relationship status

When stratified by level of social support (Table 4), moderate or high levels of controlling behavior were associated with 2.4 (95% CI 1.2, 4.9) and 1.7 (95% CI 1.0, 2.7) times the prevalence of depressive symptoms among those with low and high social support, respectively. When stratified by level of social support, emotional and physical IPV were associated with greater prevalence of depressive symptoms among individuals with low social support (emotional IPV: aPR 1.9 [95% CI 1.0, 3.4]; physical IPV: aPR 1.8 [95% CI 1.2, 2.8]), but not among those with high social support (emotional IPV: aPR 1.0 [95% CI 0.6, 1.6]; physical IPV: aPR 1.0 [95% CI 0.6, 1.6]). Among those with low social support, moderate or high levels of controlling behavior were associated with 0.9 (95% CI 0.5, 1.6) times the prevalence of hazardous alcohol use, while among those with high social support, moderate or high levels of controlling behavior were associated with 1.4 (95% CI 1.1, 1.8) times the prevalence of hazardous alcohol use. When stratified by level of social support, emotional IPV was associated with 1.4 (95% CI 0.8, 2.5) and 1.6 (95% CI 1.2, 2.0) times the prevalence of hazardous alcohol use among those with low and high social support, respectively. When stratified by level of social support, physical IPV was associated with 1.1 (95% CI 0.6, 2.0) and 1.3 (95% CI 1.0, 1.7) times the prevalence of hazardous alcohol use among those with low and high social support, respectively. Sexual IPV was not associated with prevalence of depressive symptoms or hazardous alcohol use among those with low or high social support.

**Table 4:** Multivariable models of IPV, depressive symptoms, and hazardous alcohol use among PWH initiating care in Cameroon, stratified by social support (*n* = 403).

	Depressive Symptoms				Hazardous Alcohol Use			
	Low Social Support (*n* = 98)		High Social Support (*n* = 305)		Low Social Support (*n* = 98)		High Social Support (*n* = 305)	
	aPR (95% CI)*	p-value	aPR (95% CI)*	p-value	aPR (95% CI)*	p-value	aPR (95% CI)*	p-value
Controlling Behavior	2.43 (1.21, 4.88)	0.013	1.65 (0.99, 2.74)	0.056	0.90 (0.52, 1.55)	0.710	1.41 (1.09, 1.81)	0.008
Emotional IPV	1.88 (1.03, 3.43)	0.040	1.01 (0.65, 1.57)	0.961	1.45 (0.84, 2.53)	0.186	1.57 (1.25, 1.97)	<0.001
Physical IPV	1.84 (1.21, 2.8)	0.004	1.00 (0.61, 1.63)	0.991	1.07 (0.57, 2.01)	0.834	1.29 (1.00, 1.67)	0.047
Sexual IPV	1.42 (0.77, 2.64)	0.265	1.21 (0.73, 2.00)	0.456	1.37 (0.78, 2.38)	0.269	1.15 (0.86, 1.54)	0.346

aPR: adjusted prevalence ratio

*Adjusted for gender, clinic, and relationship status

## Discussion

This research estimated the prevalence of IPV among PWH initiating HIV care in Cameroon, the relationships among IPV, depression, and hazardous alcohol use, and the extent to which these relationships varied by level of social support. In this sample, IPV was commonly reported, with controlling behavior and emotional IPV the most frequent types of IPV reported. Emotional and physical IPV were associated with greater prevalence of depressive symptoms among those with low social support, but not among those with high social support. Controlling behavior was also associated with greater prevalence of depressive symptoms, and this relationship did not meaningfully differ by level of social support. Emotional IPV, physical IPV, and controlling behavior were associated with greater prevalence of hazardous alcohol use, with moderately larger effect estimates among those with high compared to low social support. Sexual IPV was not associated with depressive symptoms or hazardous alcohol use in this group of PWH in Cameroon.

IPV was commonly reported among this group of PWH in Cameroon. Overall, 54% of this sample (46% of men and 59% of women) experienced emotional, physical, or sexual IPV in the past 12 months. This is higher than previous estimates of IPV reported among the general population in Cameroon, which estimated that 22% of men and 32% of women had experienced emotional, physical, or sexual IPV in the past 12 months [44]. The prevalence of experiencing any controlling behavior in our sample was higher than previous estimates in Cameroon. Overall, 55% of study participants (53% of men and 56% of women) reported experiencing 2 or more types of controlling behavior. Previous estimates of controlling behavior in Cameroon classify controlling behavior as endorsing at least one type of controlling behavior; in our sample, 78% of men and 79% of women endorsed at least one type of controlling behavior, which is higher than previous estimates among men (70%) and women (62%) in the Cameroon general population [44]. Estimates of emotional and sexual IPV were also higher in this study compared to previous estimates among the general Cameroonian population and among women with HIV in Cameroon. Overall, 42% of study participants reported having experienced emotional IPV (38% of men and 45% of women). Previous research found that in the 12 months prior to survey, emotional IPV was reported by 18% of men and 22% of women in the general Cameroonian population [44] and 29% of women with HIV in Cameroon [8]. Overall, 24% of study participants reported having experienced sexual IPV (22% of men and 25% of women), which is higher than previous estimates of sexual IPV in the Cameroon general population (4% of men and 7% of women) [44] and among women with HIV in Cameroon (18%) [8]. The prevalence of physical IPV in this study was 28% overall (20% of men, and 34% of women). These findings were similar to previous estimates of physical IPV among the general population of Cameroonian women (20%) [44] and among women with HIV in Cameroon (22%) [8], though higher than previous estimates of the general population of men (7%) in Cameroon [44]. The authors are not aware of previous estimates of physical IPV among men with HIV in Cameroon. It is unclear why estimates of controlling behavior, emotional IPV, and sexual IPV were higher among study participants than previous estimates. Additional research is needed to better understand the prevalence of IPV among PWH, especially among men with HIV in Cameroon and other resource-constrained countries, as research into the prevalence and impact of IPV among men with HIV in Cameroon and throughout sub-Saharan Africa remains limited.

Given the high prevalence of IPV reported by this group of PWH among both men and women, services to screen for and respond to IPV among PWH in Cameroon are urgently needed and should be integrated into HIV care settings, particularly as previous studies have demonstrated high acceptability of IPV screening in healthcare settings [49, 50]. Implementation science research to identify feasible and sustainable strategies to integrate IPV screening and support services into HIV care settings for all genders should be prioritized. In addition, it is important to consider how the needs of those experiencing bidirectional violence (i.e., both experiencing and perpetrating IPV) might be different, as couples affected by bidirectional violence may have an especially high risk of developing mental health disorders [51]. Research into effective strategies to prevent IPV among PWH and their partners is needed, particularly as evidence remains equivocal on the effectiveness of interventions to prevent IPV and has been found to vary by gender, intervention approach, setting, and the type of IPV measured [22, 52–59].

Emotional and physical IPV were associated with greater prevalence of depressive symptoms among those with low social support, but not among those with high social support. Controlling behavior was also associated with greater prevalence of depressive symptoms. However, this relationship did not vary meaningfully by level of social support. The authors are not aware of previous research that has examined the relationships among IPV, depression, and social support among PWH in sub-Saharan Africa. However, prior research focused on IPV and depression has consistently found IPV to be associated with increased prevalence of depression among general populations and among PWH [60–62]. Additional research into the relationships among IPV, depression and social support among PWH is needed. Longitudinal research that examines the mechanisms through which social support, depression, and IPV interact may provide useful insights. Interventions for those experiencing emotional or physical IPV should consider incorporating components to bolster social support. Previous research has demonstrated psychotherapeutic group support interventions to be feasible and acceptable in sub-Saharan African settings [63] and effective in reducing depressive symptoms among pregnant women with HIV [64]. A systematic review of IPV interventions focused on improving social support or mental health for survivors of IPV found that most interventions resulted in improved social support or mental health despite little evidence of reductions in IPV [65]. The extent to which such interventions are effective and appropriate with PWH in Cameroon warrants further investigation. Future research should examine strategies to support the mental health of PWH experiencing controlling behavior, particularly as strategies to enhance social support may be insufficient to manage depressive symptoms among PWH experiencing controlling behaviors.

Integrated interventions that address both IPV and depression should be developed, implemented, and evaluated with PWH. A review of interventions to improve the mental health of women who have experienced IPV found that psychosocial interventions that were holistic, individualized, and trauma-informed were more likely to be associated with improved mental health [66]. While few integrated IPV and mental health interventions have been developed to date [67], cognitively and behaviorally-based interventions have been associated with improved mental health among women who have experienced IPV [57, 68, 69]. However, these types of interventions often require significant human resources and capacity building and may be challenging to implement in resource-constrained settings. Feasible and sustainable strategies to integrate such strategies into resource-constrained settings should be identified.

Emotional IPV, physical IPV, and controlling behavior were associated with greater prevalence of hazardous alcohol use, with moderately larger effect estimates among those with high compared to low social support. Previous research has established a positive association between both physical and sexual IPV and alcohol use in longitudinal and cross-sectional studies in both the general population and among PWH [21, 23, 70, 71]. However, limited research and varying definitions of emotional IPV across studies have presented challenges to characterizing the relationship between emotional IPV and alcohol use [21]. Little is known about the relationships among IPV, alcohol, and social support, particularly among PWH in sub-Saharan Africa. The relationships among hazardous alcohol use, IPV, and social support warrant greater exploration among PWH throughout sub-Saharan Africa. Few evidence-based interventions exist that address both IPV and hazardous alcohol use. However, an RCT of a couples-based Common Elements Treatment Approach found that the intervention was associated with reduced IPV and hazardous alcohol use among high-risk couples with reductions sustained up to two years post-intervention [72, 73]. In addition, HIV status moderated intervention effectiveness with greater reductions in IPV among women living with compared to without HIV [68]. The feasibility and appropriateness of integrating such an intervention into HIV care should be explored.

Sexual IPV was not associated with depressive symptoms or hazardous alcohol use. This is contrary to previous research which found sexual IPV to be associated with increased prevalence of depression and hazardous alcohol use. A prospective study among women living with HIV in rural Uganda that found sexual IPV to be associated with increased risk of depression and heavy drinking [12]. Additional research is needed to better understand the relationship between sexual IPV, depression, and alcohol use among PWH in Cameroon. Examining potential mediators and moderators of the relationship between sexual IPV and depression and alcohol use may provide useful insights.

Controlling behavior was associated with depressive symptoms and hazardous alcohol use. Research on controlling behavior remains limited, particularly in sub-Saharan Africa and among PWH. However, a study among women who recently gave birth in Rwanda found controlling behavior had the strongest association with depression, among all forms of IPV assessed [74]. Evidence on interventions to address controlling behavior is mixed. Improving couple communication has shown promise in reducing controlling behaviors by male partners [75]. However, an intervention to address IPV and economic security by transforming gender attitudes and strengthening livelihoods did not meaningfully impact men’s controlling behavior [53]. Future research is needed to advance understanding of the relationship between controlling behavior and mental health and identify effective interventions to prevent controlling behavior and improve the mental health of individuals experiencing controlling behavior in intimate relationships.

Several limitations in this study are worth noting. First, data were cross-sectional. Thus, the temporal relationships among IPV, depressive symptoms, and hazardous alcohol use cannot be established. Previous research demonstrates bidirectional pathways between IPV and depression [22, 76] and IPV and substance use [77, 78]. Longitudinal research is needed to understand causal pathways among these variables. Second, this study was conducted with PWH initiating HIV care in three HIV clinics in Cameroon. Findings may differ among PWH from other regions in Cameroon and among PWH at other points in the HIV care continuum. Third, due to the sensitive nature of the questions in the survey, there is a potential for underreporting. To minimize underreporting, interviewers were rigorously trained to build rapport, ensure confidentiality, and employ empathetic communication strategies. Fourth, HIV disclosure has been associated with IPV among PWH in sub-Saharan Africa [79]. As HIV disclosure information was not available for this study population, we are unable to assess the role of HIV disclosure in the relationships among IPV, depression, and alcohol use. Finally, there are limitations in the measures used in this study. The social support measure used in this analysis included 4 of 12 items from the MSPSS. The psychometric properties of the NDHS domestic violence module among PWH in Cameroon should be investigated further [45]. Despite being previously validated for use in sub-Saharan Africa, PHQ-9 cutoff scores for moderate depression vary widely depending on context [80]. While the PHQ-9 has been validated in Cameroon with PWH it demonstrated high specificity but low sensitivity [37].

## Conclusion

This paper explores the interrelationships among IPV, depression, hazardous alcohol use, and social support among PWH initiating HIV care in Cameroon. Our findings suggest services to screen and care for people experiencing IPV are urgently needed in this population, and more attention should be given to the impact controlling behavior has on mental health. Given the diverse relationships depending on the type of IPV experienced and level of social support, a one-size-fits-all approach to IPV prevention and response programming among PWH may be inadequate. Future implementation research can identify the potential barriers, feasibility, acceptability, and organizational readiness to integrate IPV and mental health into HIV services in clinical settings and identify needed resources for success.

## Supporting information

S1 Checklist Inclusivity in global research. (DOCX)
